# Supplementary material for: Investigation of Excited-State Intramolecular Proton Transfer and Structural Dynamics in Bis-Benzimidazole Derivative (BBM)
Source: Int J Mol Sci. 2023 May 29;24(11):9438. doi: 10.3390/ijms24119438 (PMC10253469; doi:10.3390/ijms24119438)
Supplement: Supplementary file 1 [file ijms-24-09438-s001.zip › ijms-2388929-supplementary.pdf]

Investigation of Excited State Intramolecular Proton Transfer and Structural Dynamics in Bis-benzimidazole Derivative (BBM)

Junhan Xie [1], Ziyu Wang [1], Ruixue Zhu [1], Jiaming Jiang [1], Tsu-Chien Weng [1], Yi Ren [1], Shuhua Han [3], Yifan Huang [1,*] and Weimin Liu [1,2,*]

[1] School of Physical Science and Technology, ShanghaiTech University, Shanghai 201210, China
[2] STU and SIOM Joint Laboratory for Superintense Lasers and the Applications, Shanghai 201210, China
[3] Key Lab of Colloid and Interface Chemistry, Ministry of Education, Shandong University, Jinan 250100, China

[†]*These authors contributed equally to this work.*

* *To whom correspondence should be addressed. E-mails:* *liuwm@shanghaitech.edu.cn*

huangyf@shanghaitech.edu.cn.

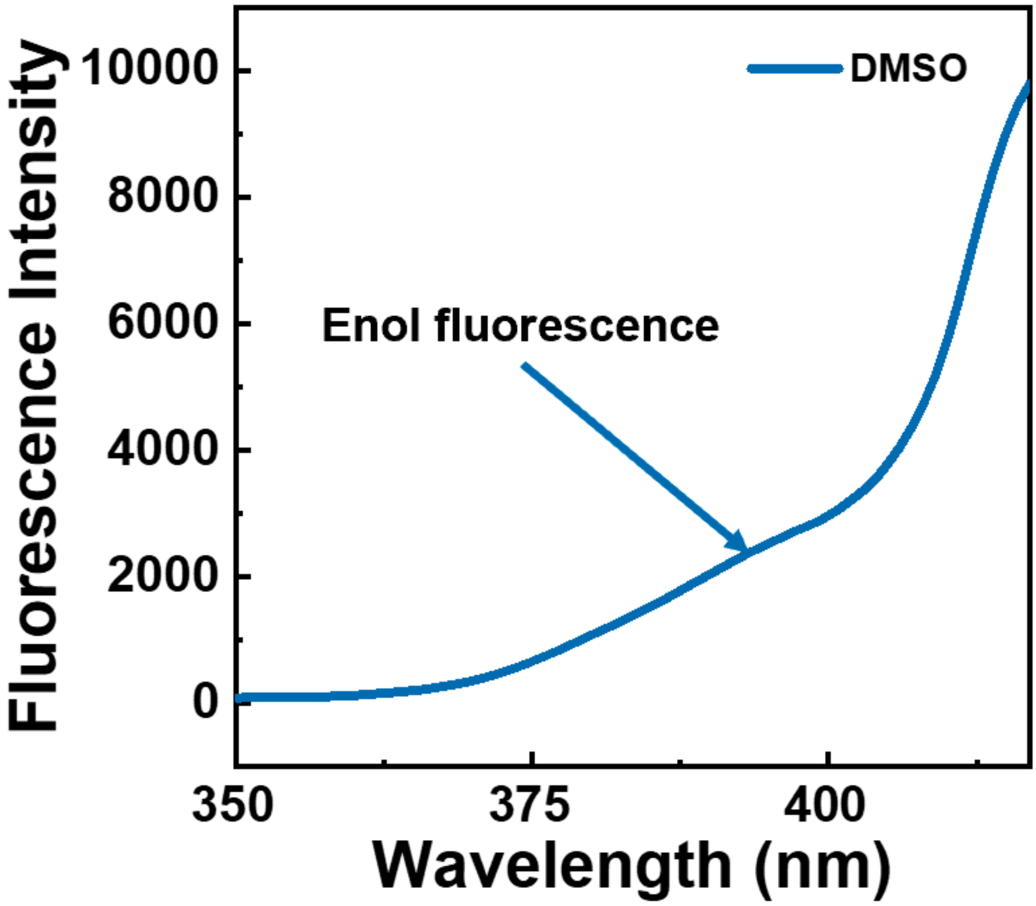

Figure S1. Steady-state fluorescence spectra of HBT in DMSO in the range of 350 to 430 nm.

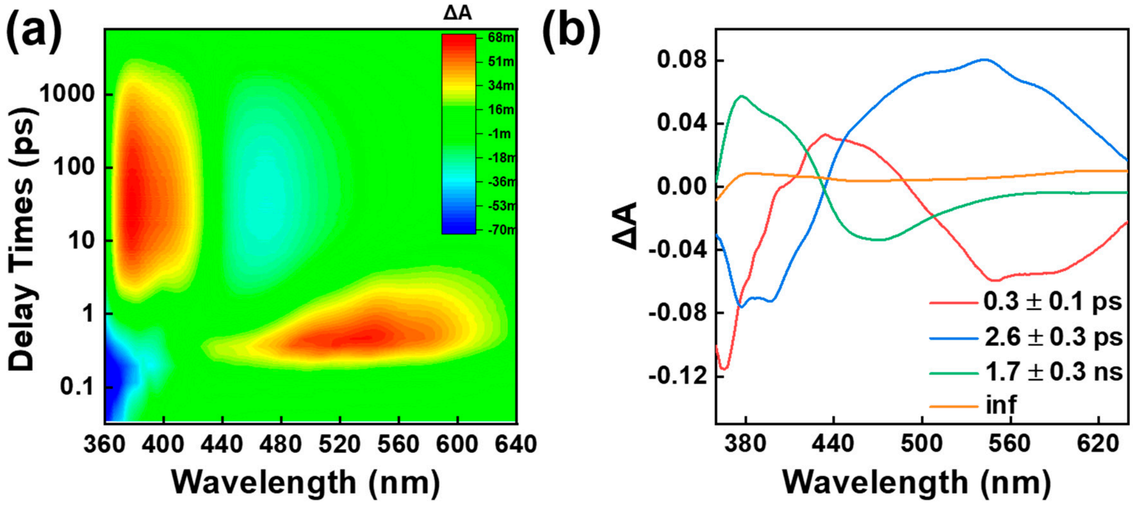

Figure S2. (a) 2D plot of TA spectrum of BBM in THF with 350 nm excitation (time delay is plotted using log scale). (b) Decay-associated difference spectra (DADS) of TA spectrum of BBM in THF.

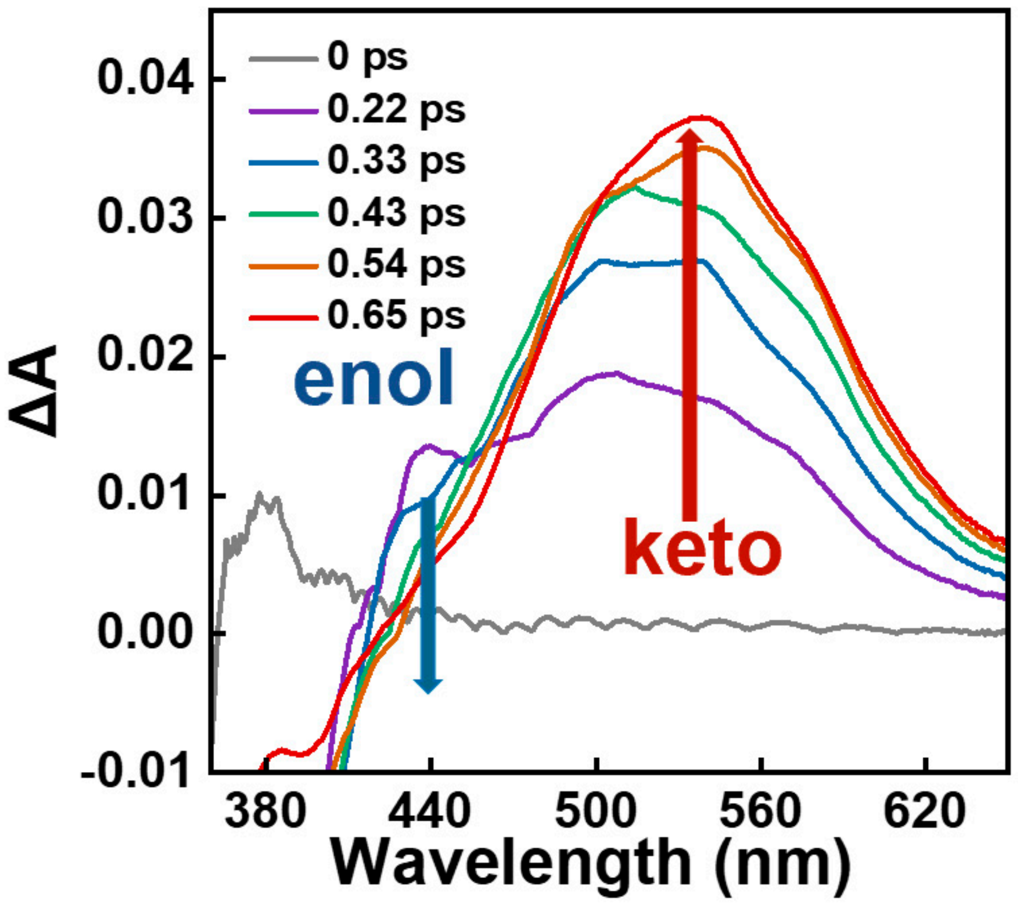

Figure S3. TA spectra of BBM in DMSO at different delay times between 0 and 0.65 ps.

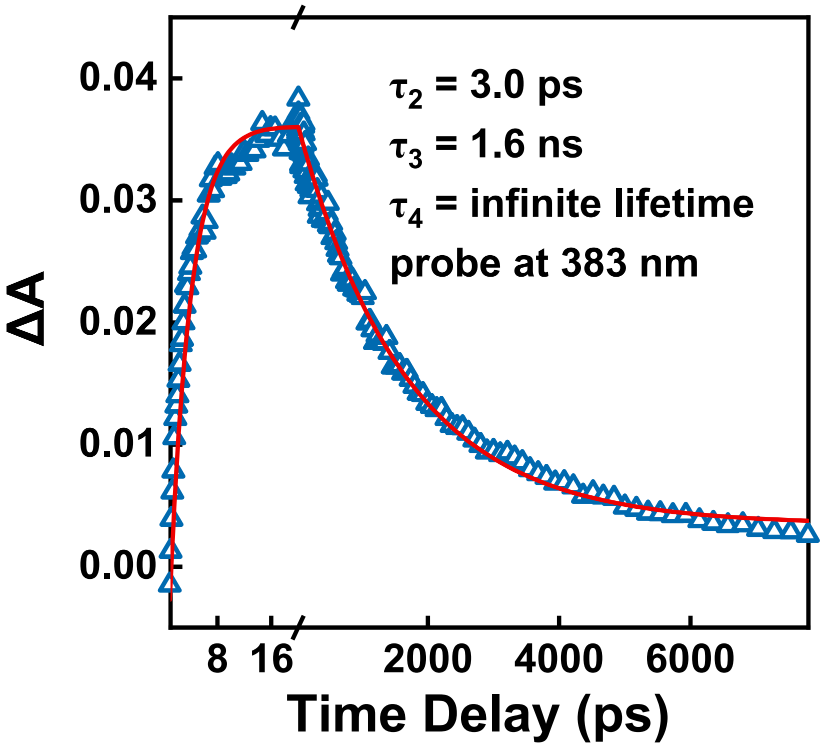

Figure S4. Transient amplitude of the TA spectra of BBM probe at 383 nm in DMSO.

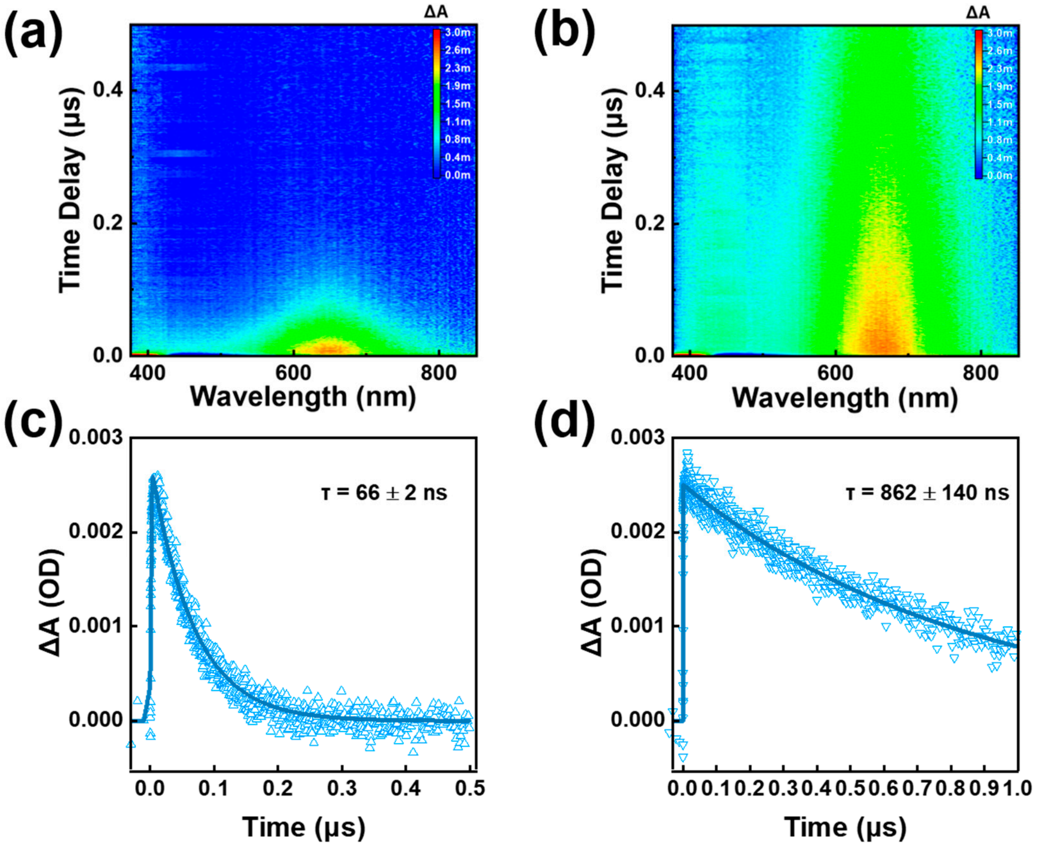

Figure S5. 2D plot of EOS-TA of BBM in (a) DMSO, and (b) THF, transient amplitude of the EOS-TA spectra of BBM probe at 667 nm in (c) DMSO, and (d) THF.

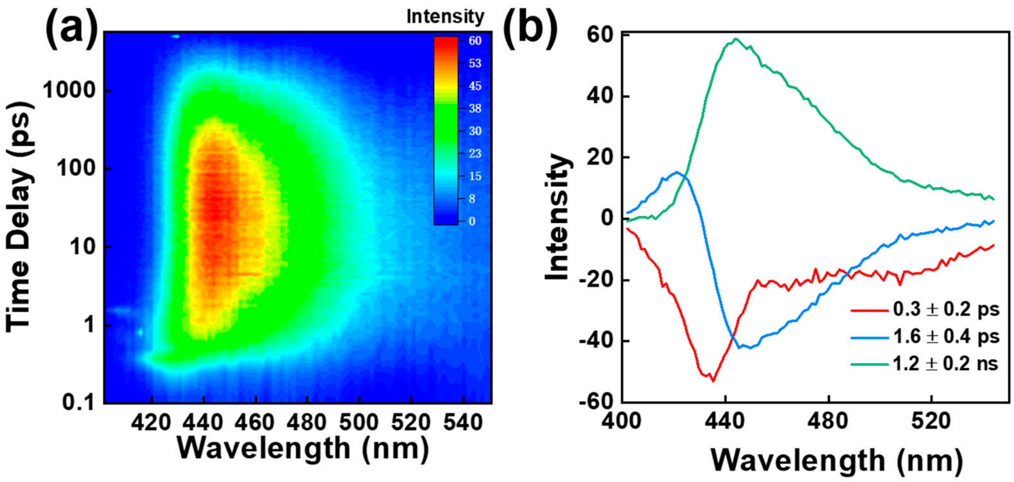

Figure S6. (a) 2D plot of fs fluorescence up-conversion spectra of BBM under 350 nm excitation in THF (time delay is plotted using log scale); (b) DADS of fs fluorescence up-conversion spectra of BBM in THF.

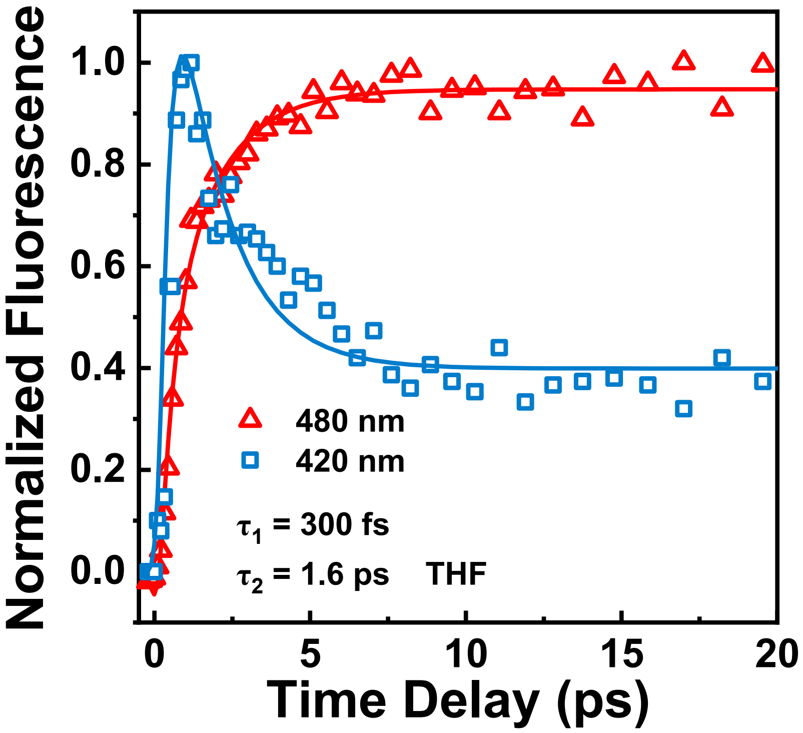

Figure S7. Transient amplitude of BBM-keto* fluorescence probe at 420 nm and 480 nm in THF， τ_1 and τ_2 are retrieved from global analysis.

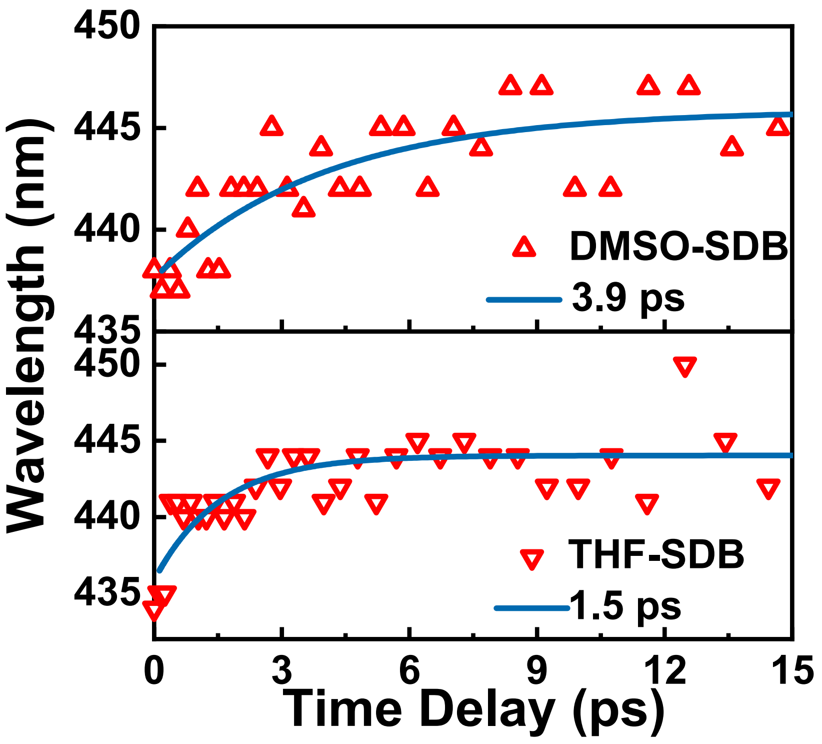

Figure S8. Transient fluorescence frequency shift of BBM-keto* in DMSO and THF.

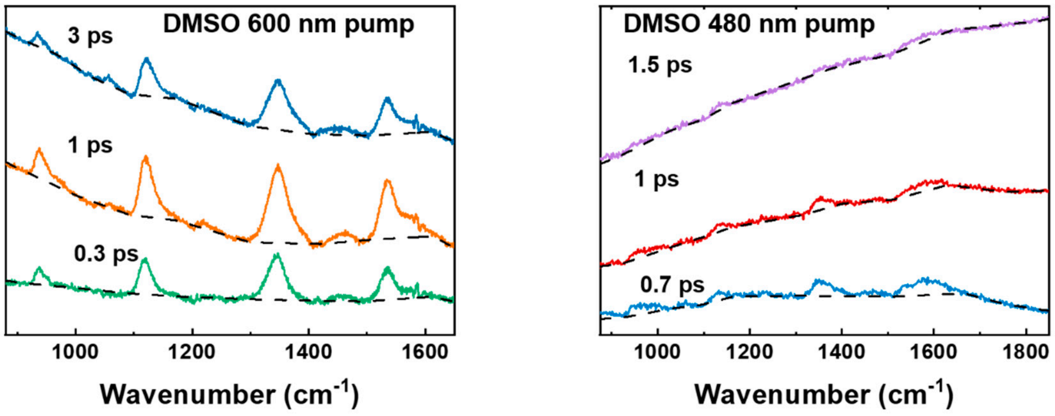

Figure S9. Excited state stimulated Raman data of BBM at different time delays with the baseline drawn (dash line).

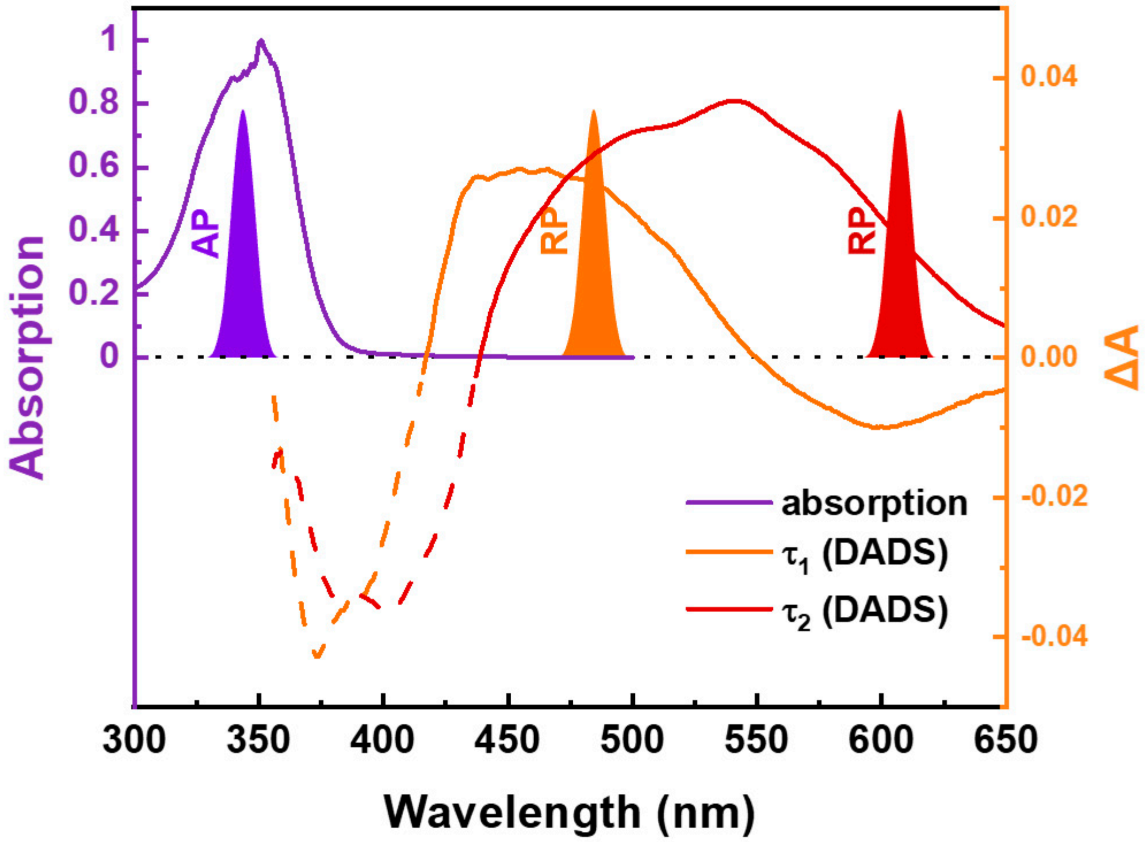

Figure S10. Steady-state absorption spectrum and DADS spectrum of τ_1 and τ_2 in TA. The actinic pump (AP) at 350 nm and Raman pump (RP) at 480 and 600 nm are depicted by colored spikes.

Enol

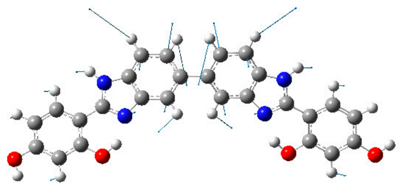

Mode (I)

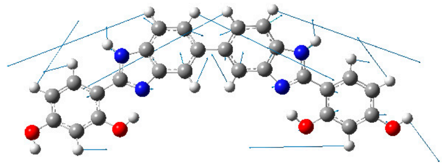

Mode (II)

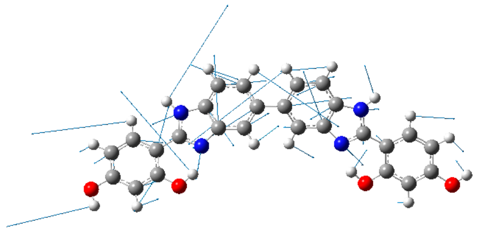

Mode (III)

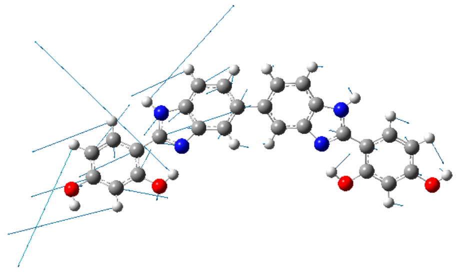

Mode (IV)

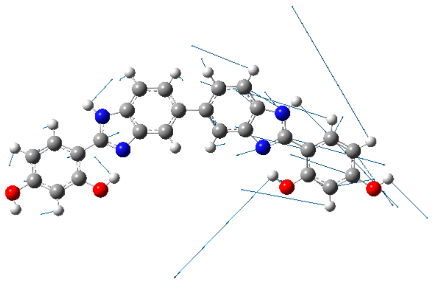

Mode (V)

Figure S11. DFT calculation of BBM-Enol form showing optimized structure and Raman modes (I-V).

Keto-pt2

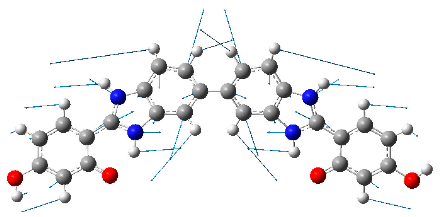

Mode(I)

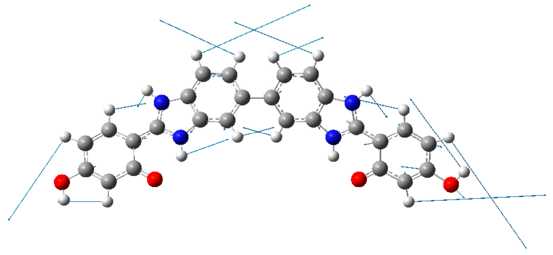

Mode(II)

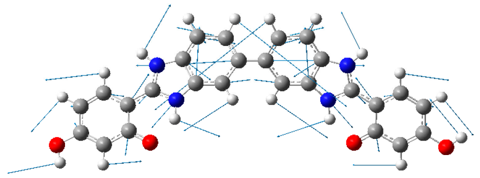

Mode(III)

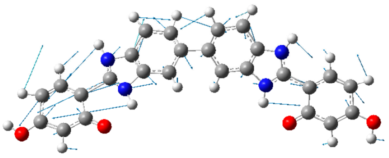

Mode(IV)

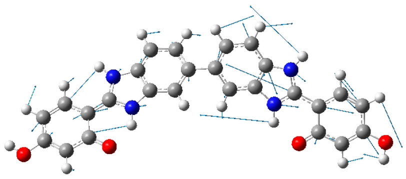

Mode(V)

Figure S12. DFT calculation BBM-Keto form (ESIPT occurs in both HBI halves) showing optimized structure and Raman modes (I-V).

Keto-pt1

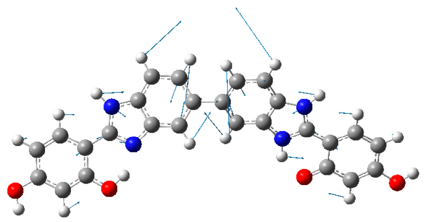

Mode(I)

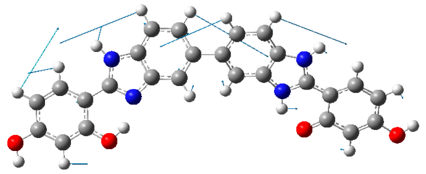

Mode(II)

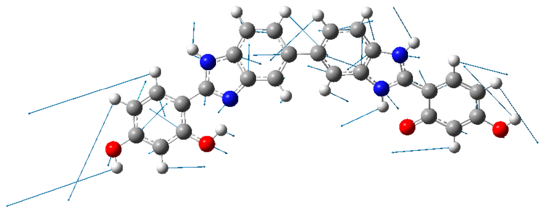

Mode(III)

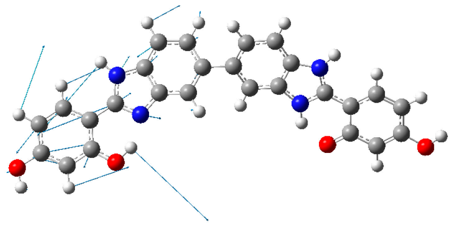

Mode(IV)

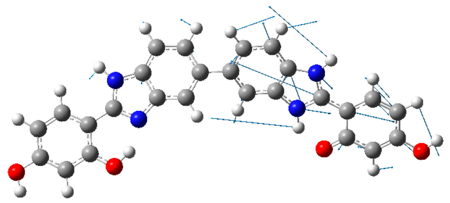

Mode(V)

Figure S13. DFT calculation (ESIPT occurs in one HBI halve) showing optimized structure and Raman modes (I-V).

Table S1. Detailed description of Raman modes was achieved through experiment (exp) and DFT calculations (theo).

		enol	keto-pt2[a]	keto-pt1[b]	Vibrational mode assignment
(I)	theo.	960	959	973	C-H bending out of plane
	exp.	953		939	
(II)	theo.	1160	1166	1163	C-H waging in plane
	exp.	1130		1122	
(III)	theo.	1335	1343	1369	Benzene and imidazole rings stretching and C-H in plane waging
	exp.	1358		1348	
(IV)	theo.	1566	1601	1595	C-C stretching
	exp.	1565		1534	
(V)	theo.	1570	1604	1633	C-C stretching
	exp.	1587		1568	

[a] DFT results for ESIPT occurred in one HBI half of BBM-keto form

[b] DFT results for ESIPT occurred in two HBI halves of BBM-keto form

Table S2. Atomic coordinates of Enol Keto-pt2 and Keto-pt1 by DFT calculation.

Enol

Tag	Symbol	X	Y	Z
1	C	-2.87443	0.722347	-0.00211
2	C	-3.49094	1.883843	-0.51372
3	C	-2.75829	3.026157	-0.82667
4	C	-1.38525	2.975014	-0.61233
5	C	-0.73776	1.824311	-0.09883
6	C	-1.49484	0.68923	0.209934
7	C	-5.0009	0.290679	-0.13694
8	H	-3.22829	3.915454	-1.22875
9	H	-0.78818	3.841873	-0.86866
10	H	-1.02984	-0.19646	0.625857
11	H	-5.56566	2.193726	-0.91548
12	C	0.737179	1.822852	0.103209
13	C	1.387176	2.972867	0.615159
14	C	1.491895	0.685998	-0.20475
15	H	0.791869	3.841108	0.870924
16	C	2.871748	0.716848	0.006131
17	H	1.025001	-0.19921	-0.61956
18	C	3.490759	1.877859	0.515759
19	C	2.760427	3.021792	0.828338
20	H	3.232357	3.910713	1.228992
21	C	4.997625	0.282072	0.137978
22	H	5.566324	2.185258	0.913785
23	C	6.263923	-0.4275	0.052882
24	C	8.684112	-0.52939	0.345805
25	C	7.493278	-2.45998	-0.51352
26	C	8.681608	-1.84816	-0.13032
27	H	9.611587	-0.05406	0.643799
28	H	7.496814	-3.47864	-0.88074
29	C	-6.26768	-0.4177	-0.05481
30	C	-6.29046	-1.75367	0.425871
31	C	-7.48907	0.166099	-0.43673
32	C	-7.49968	-2.44925	0.507515
33	C	-8.68737	-0.51929	-0.35766
34	H	-7.50809	1.184238	-0.80707
35	C	-8.68658	-1.83802	0.11817
36	H	-7.49	-3.46816	0.878121
37	H	-9.61931	-0.05682	-0.65602
38	O	-9.88876	-2.47837	0.178245
39	H	-9.77461	-3.37629	0.513524
40	O	-5.17235	-2.39827	0.816274
41	H	-4.40238	-1.77422	0.698158
42	O	9.824684	-2.5839	-0.23969
43	H	10.58393	-2.06453	0.051968
44	O	5.165138	-2.40907	-0.81308

**45**	H	4.396646	-1.78431	-0.69362
**46**	N	-3.83857	-0.24578	0.223194
**47**	N	-4.83818	1.576661	-0.58892
**48**	N	4.83754	1.568681	0.589269
**49**	N	3.834092	-0.253	-0.21962
**50**	C	6.286175	-1.76567	-0.42767
**51**	C	7.484358	0.156721	0.429872
**52**	H	7.505886	1.174817	0.799962

Keto-pt2

Tag	Symbol	X	Y	Z
**1**	C	-2.86143	0.71999	-0.00235
**2**	C	-3.48992	1.868822	-0.51168
**3**	C	-2.75852	3.008485	-0.82501
**4**	C	-1.38392	2.959773	-0.61137
**5**	C	-0.7363	1.813634	-0.09855
**6**	C	-1.49013	0.673428	0.213043
**7**	C	-5.0629	0.299366	-0.15038
**8**	H	-3.23151	3.895216	-1.22747
**9**	H	-0.7896	3.827326	-0.86997
**10**	H	-1.02644	-0.21148	0.629677
**11**	H	-5.56413	2.197932	-0.91966
**12**	C	0.736979	1.812515	0.104014
**13**	C	1.386748	2.958508	0.614791
**14**	C	1.488796	0.670837	-0.20653
**15**	H	0.793849	3.82724	0.87268
**16**	C	2.860489	0.715819	0.007253
**17**	H	1.023505	-0.21395	-0.62161
**18**	C	3.49107	1.86471	0.513989
**19**	C	2.761537	3.005767	0.826846
**20**	H	3.236134	3.892499	1.22741
**21**	C	5.061139	0.293391	0.151511
**22**	H	5.566479	2.192135	0.917457
**23**	C	6.284304	-0.42873	0.054039
**24**	C	8.700628	-0.54223	0.34341
**25**	C	7.494828	-2.47735	-0.52359
**26**	C	8.673525	-1.87243	-0.14198
**27**	H	9.637952	-0.08416	0.637488
**28**	H	7.504757	-3.4966	-0.89172
**29**	C	-6.28712	-0.41991	-0.0566
**30**	C	-6.24877	-1.78614	0.448644
**31**	C	-7.51783	0.160918	-0.442
**32**	C	-7.50607	-2.46413	0.517308
**33**	C	-8.70375	-0.5279	-0.35898
**34**	H	-7.54163	1.17885	-0.816
**35**	C	-8.68153	-1.85693	0.127716

**36**	H	-7.50739	-3.4836	0.889754
**37**	H	-9.64362	-0.08079	-0.65519
**38**	O	-9.88949	-2.49287	0.185953
**39**	H	-9.77393	-3.3895	0.524729
**40**	O	-5.16193	-2.35584	0.812142
**41**	H	-3.87642	-1.18563	0.56101
**42**	O	9.825342	-2.60166	-0.24746
**43**	H	10.57764	-2.07493	0.048359
**44**	O	5.149647	-2.36268	-0.80654
**45**	H	3.875421	-1.19274	-0.55509
**46**	N	-3.86499	-0.21369	0.206483
**47**	N	-4.84664	1.570188	-0.5908
**48**	N	4.847628	1.5647	0.590927
**49**	N	3.862602	-0.21919	-0.20194
**50**	C	6.241764	-1.79679	-0.44968
**51**	C	7.515662	0.150527	0.432405
**52**	H	7.5447	1.168756	0.805034

Keto-pt1

Tag	Symbol	X	Y	Z
**1**	C	2.858132	0.719015	0.001202
**2**	C	3.473547	1.880377	0.504553
**3**	C	2.745465	3.022936	0.81335
**4**	C	1.376253	2.971291	0.6019
**5**	C	0.732277	1.820818	0.097014
**6**	C	1.481703	0.684932	-0.20712
**7**	C	4.97386	0.294335	0.1359
**8**	H	3.217719	3.914047	1.21112
**9**	H	0.777005	3.839098	0.855879
**10**	H	1.013256	-0.20254	-0.61891
**11**	H	5.54122	2.192467	0.900671
**12**	C	-0.73541	1.820458	-0.10175
**13**	C	-1.3846	2.966829	-0.60165
**14**	C	-1.4825	0.679295	0.200836
**15**	H	-0.78945	3.837687	-0.85357
**16**	C	-2.85162	0.724418	-0.01149
**17**	H	-1.01334	-0.20804	0.609624
**18**	C	-3.48175	1.874462	-0.50688
**19**	C	-2.75564	3.016451	-0.81098
**20**	H	-3.23206	3.90685	-1.20407
**21**	C	-5.03752	0.308278	-0.15561
**22**	H	-5.55144	2.203566	-0.90427
**23**	C	-6.25161	-0.42402	-0.05674
**24**	C	-8.65976	-0.56768	-0.32708
**25**	C	-7.41969	-2.48438	0.516152
**26**	C	-8.60942	-1.89638	0.148683

**27**	H	-9.60695	-0.12088	-0.61105
**28**	H	-7.41281	-3.50682	0.878648
**29**	C	6.23352	-0.41505	0.055085
**30**	C	6.242652	-1.74938	-0.41888
**31**	C	7.454503	0.162773	0.430727
**32**	C	7.445527	-2.45006	-0.49945
**33**	C	8.645226	-0.52802	0.352493
**34**	H	7.479798	1.183807	0.7968
**35**	C	8.634802	-1.84659	-0.11626
**36**	H	7.428683	-3.47175	-0.86565
**37**	H	9.582028	-0.07043	0.646827
**38**	O	9.822398	-2.49057	-0.17549
**39**	H	9.696932	-3.38636	-0.5044
**40**	O	5.126029	-2.37885	-0.79946
**41**	H	4.365488	-1.74098	-0.67799
**42**	O	-9.74007	-2.6372	0.258515
**43**	H	-10.4975	-2.11773	-0.02774
**44**	N	3.816682	-0.24499	-0.21956
**45**	N	4.813243	1.574995	0.578761
**46**	N	-4.83121	1.576093	-0.58382
**47**	C	-6.18254	-1.78745	0.435899
**48**	C	-7.48747	0.140597	-0.42085
**49**	H	-7.53192	1.162159	-0.78673
**50**	N	-3.84738	-0.20769	0.190822
**51**	H	-3.88779	-1.19295	0.53584
**52**	O	-5.0816	-2.33582	0.779076

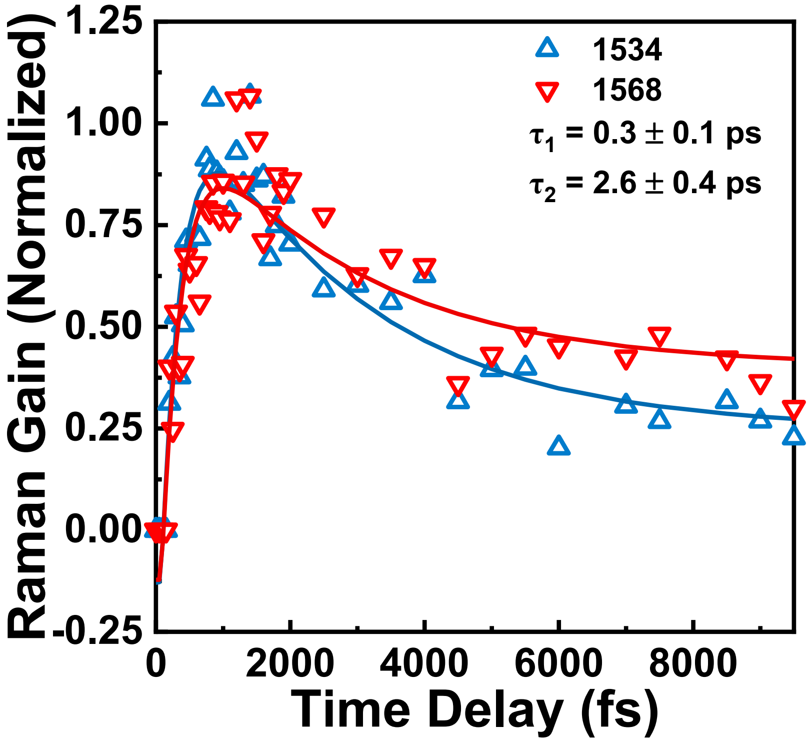

Figure S14. Transient amplitude Raman modes IV and V of BBM-keto* in THF with 600 nm Raman pump excitation.

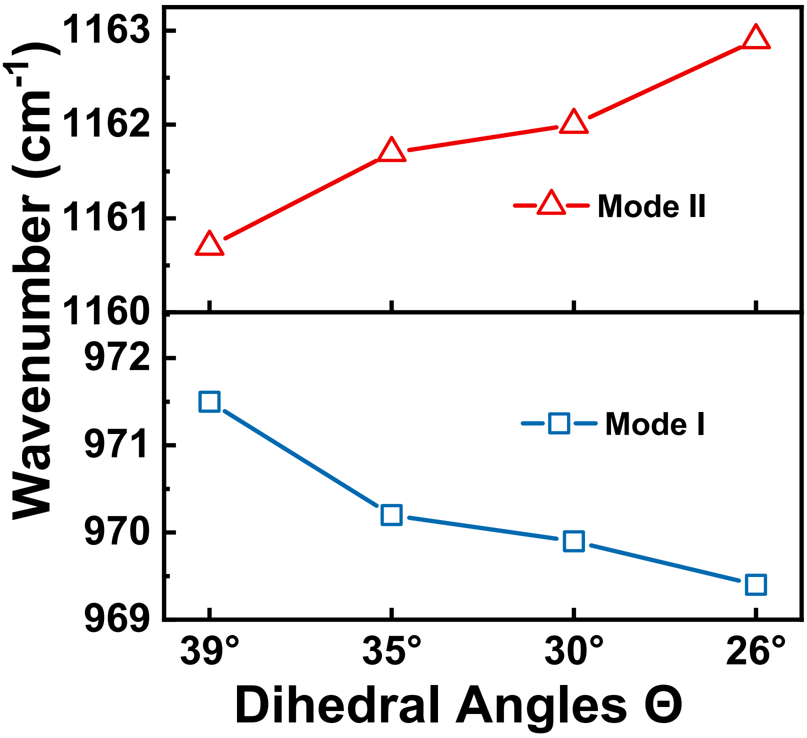

Figure S15. Transient frequency shift of BBM-keto* in TD-DFT calculation.

Table S3. TD-DFT calculation of emission wavelength and oscillator strength of BBM-keto* at $\Theta = 35°$, $30°$, and $26°$.

Θ	35°	30°	26°
Emission wavelength (nm)	419	420	421
Oscillator strength	0.97	1.01	1.05

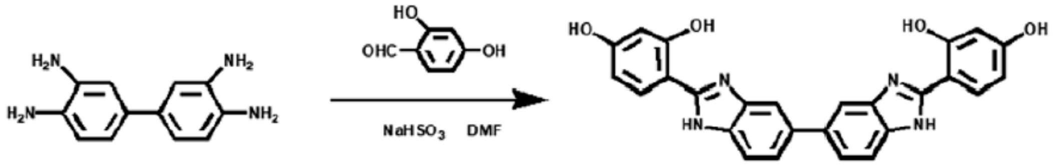

Scheme S1. Synthetic route of bis-benzimidazole derivative [26].
